# Transient resting-state salience-limbic co-activation patterns in functional neurological disorders

**DOI:** 10.1016/j.nicl.2024.103583

**Published:** 2024-02-28

**Authors:** Samantha Weber, Janine Bühler, Serafeim Loukas, Thomas A.W. Bolton, Giorgio Vanini, Rupert Bruckmaier, Selma Aybek

**Affiliations:** a Department of Neurology, Psychosomatic Medicine Unit, Inselspital Bern University Hospital, University of Bern, 3012 Bern, Switzerland; b University of Zurich, Psychiatric University Hospital Zurich, Department of Psychiatry, Psychotherapy and Psychosomatics, 8032 Zurich, Switzerland; c Translational Imaging Center (TIC), Swiss Institute for Translational and Entrepreneurial Medicine, 3010 Bern, Switzerland; d Institute of Bioengineering, Ecole Polytechnique Fédérale de Lausanne (EPFL), 1015 Lausanne, Switzerland; e Division of Development and Growth, Department of Pediatrics, University of Geneva, 1211 Geneva, Switzerland; f Department of Clinical Neurosciences, Neurosurgery Service and Gamma Knife Center, Centre Hospitalier Universitaire Vaudois, 1011 Lausanne, Switzerland; g Department of Radiology, Centre Hospitalier Universitaire Vaudois, 1011 Lausanne, Switzerland; h Veterinary Physiology, Vetsuisse Faculty, University of Bern, 3012 Bern, Switzerland; i Faculty of Science and Medicine, University of Fribourg, 1700 Fribourg, Switzerland

**Keywords:** Conversion disorders, Dynamic functional connectivity, Interoception, Biomarker, Co-activation pattern, Stress

## Abstract

**Background:**

Functional neurological disorders were historically regarded as the manifestation of a *dynamic* brain lesion which might be linked to trauma or stress, although this association has not yet been directly tested yet. Analysing large-scale brain network dynamics at rest in relation to stress biomarkers assessed by salivary cortisol and amylase could provide new insights into the pathophysiology of functional neurological symptoms.

**Methods:**

Case-control resting-state functional magnetic resonance imaging study of 79 patients with mixed functional neurological disorders (i.e., functional movement disorders, functional seizures, persistent perceptual-postural dizziness) and 74 age- and sex-matched healthy controls. Using a two-step hierarchical data-driven neuroimaging approach, *static* functional connectivity was first computed between 17 resting-state networks. Second, *dynamic* alterations in these networks were examined using co-activation pattern analysis. Using a partial least squares correlation analysis, the multivariate pattern of correlation between altered temporal characteristics and stress biomarkers as well as clinical scores were evaluated.

**Results:**

Compared to healthy controls, patients presented with functional aberrancies of the salience-limbic network connectivity. Thus, the insula and amygdala were selected as seed-regions for the subsequent analyses. Insular co-(de)activation patterns related to the salience network, the somatomotor network and the default mode network were detected, which patients entered more frequently than controls. Moreover, an insular co-(de)activation pattern with subcortical regions together with a wide-spread co-(de)activation with diverse cortical networks was detected, which patients entered less frequently than controls. In patients, dynamic alterations conjointly correlated with amylase measures and duration of symptoms.

**Conclusion:**

The relationship between alterations in insular co-activation patterns, stress biomarkers and clinical data proposes inter-related mechanisms involved in stress regulation and functional (network) integration. In summary, altered functional brain network dynamics were identified in patients with functional neurological disorder supporting previously raised concepts of impaired attentional and interoceptive processing.

## Highlights

• Patients entered more frequently into insular-somatomotor co-activation.
• Patients entered less often into insular co-activation with subcortical regions.
• Dynamic alterations correlated with alpha-amylase and duration of illness.
• Functional alterations reflect prioritizing allostatic mechanisms due to alert.

## 1 Introduction

Patients with a functional neurological disorder (FND) (American Psychiatric Association, 2013) present with neurological symptoms (Bennett et al., 2021) that cannot be attributed to a classical neurological disease (Drane et al., 2020) but are rather of functional nature. In the late 19th century, Jean-Martin Charcot – known for his exceptional work on *hysteria*, the former name of FND – postulated that the symptoms might be produced by a functional or dynamic lesion, possibly linked to emotional trauma (Aybek, 2019). With the advances in techniques allowing to investigate brain function, studies can focus not only on *why* FND emerges, but also on *how* symptoms are produced in the absence of a neurological disease.

To answer the question on *why* FND develops, previous literature suggested that the aetiology of FND is subject to various predisposing, precipitating, and perpetuating (biopsychosocial) risk factors (Hallett et al., 2022), of which most recently an aberrant biological stress regulation has been identified (Chung et al., 2022, Weber et al., 2022) and further linked to emotional trauma (Ludwig et al., 2018, Weber et al., 2022). Despite these recent advances, it is noteworthy that a substantial portion of patients do not report on psychological stress or past trauma (Nicholson et al., 2011) and that studies investigating endocrine, immunological, or autonomic stress biomarkers in FND often suffer from limited sample sizes, subpar control cohort quality, and apply different measurement approaches, leading to difficulties in achieving replicability (Paredes-Echeverri et al., 2022).

To answer the question on *how* FND develops, comprehensive evidence from neuroimaging exists of multiple brain function alterations and network dysfunctions comprising impaired motor initiation (Cojan et al., 2009), misdirected attention (Huys et al., 2021), and abnormal interoceptive processing (Pick et al., 2019, Sojka et al., 2021). In summary, key regions involve the insula, the amygdala, the prefrontal cortex, the temporo-parietal junction (TPJ), as well as the supplementary motor area (SMA). Of particular interest are the insula and the amygdala as they might help connecting the questions on *how* and *why*. The insula represents an important hub of the salience and limbic networks, implicated in the detection of external and internal stimuli and the resulting behavioural response (Uddin, 2017). In FND, the insula has been associated with attentional and interoceptive deficits (Pick et al., 2019) and is thought to be implicated in the mediation of emotional influence on motor control (Vuilleumier, 2014). Likewise, the amygdala, a key region – amongst others – of the limbic system, is suggested to play an important role in the pathophysiology of FND. Emotional arousal and enhanced amygdalar activity have been associated with aberrant motor planning and motor behaviour (Aybek et al., 2015). Together, the insula and amygdala might directly alter motor planning and execution, and thus, bypass executive control (van der Kruijs et al., 2012).

Commonly, functional connectivity (FC) analysis of functional magnetic resonance imaging (fMRI) data in FND (Perez et al., 2021) has been assessed using *static* approaches summarizing the temporal correlation between spatially distinct brain regions, with the assumption that their interaction remains constant over time. The brain, however, is a dynamic system that constantly fluctuates between different states (Brembs, 2021). Therefore, assessing dynamic changes in FC might provide a better understanding of the fundamental properties of pathophysiological mechanisms in FND (Preti et al., 2017). Up to now, however, only two studies used a dynamic approach in FND: Diez *et al.* (Diez et al., 2019) demonstrated the potential of graph-theory step-wise FC as a prognostic biomarker for FND, whereas Marapin *et al.* (Marapin et al., 2020) studied the spatial and temporal characteristics of dynamic brain states in FND patients using a clustering approach based on sliding-window dynamic FC in brain networks derived from independent component analysis (ICA).

Here, we adopt a two-step data-driven methodological approach, in which *static* FC is first quantified to investigate network alterations in FND compared to healthy controls, to then further deepen the analyses using a *dynamic* FC approach based on co-activation pattern (CAP) analysis (Liu et al., 2018, Liu et al., 2013, Liu and Duyn, 2013).

In parallel, an urgent need remains to integrate diverse composite biomarkers including psychobiologically relevant measures into imaging studies (Perez et al., 2021, Thomsen et al., 2020). Utilizing a multifactorial and multimodal approach will contribute to advancing our understanding of the pathophysiological mechanisms in FND by bridging the gap between two lines of research: the exploration of *how* and *why*. This integration will enhance the comprehension of the interplays between stress, dynamic brain network alterations, and the manifestation of functional neurological symptoms. Therefore, in this exploratory study, we set out to expand upon the evidence gathered from the same cohort that has previously been published (Weber et al., 2022) by examining dynamic brain networks and how they relate to stress biomarkers. Pursuing this line of research might refine our understanding of FND and of how stress and functional brain alterations might interact. Therefore, we aimed at 1) identifying dynamic functional networks in healthy controls (HC) and FND patients and comparing their temporal characteristics; and 2) exploring the relationship between dynamic fMRI features, stress biomarkers, and clinical scores.

## 2 Methods

### 2.1 Participants

86 patients with mixed FND symptoms were recruited from the University Hospital Inselspital Bern, Switzerland. 76 age-and sex-matched HC were recruited through advertisement. Exclusion criteria were: 1) a current severe psychiatric disorder (*e.g.*, acute suicidality, active psychotic symptoms), 2) a major neurological (structural) disease, such as brain injuries, neurodegenerative diseases, or congenital neurological conditions, 3) alcohol or drug abuse, 4) contraindication to MRI, 5) pregnancy or breast-feeding, or 6) inadequate proficiency in the Swiss national languages to understand the study protocol and provide informed consent. The study was approved by the Ethics Committee of the Canton Bern (SNCTP000002289) and conducted according to the Declaration of Helsinki. Written informed consent was provided by all subjects. Structural imaging data, questionnaire data and cortisol data of this cohort have previously been published (Weber et al., 2022).

### 2.2 Demographic and clinical characteristics

We included FND patients with functional movement disorder (ICD-10 (World Health Organization, 1993) code F44.4) and sensory symptoms (F44.6), with functional seizures (F44.5), mixed symptom type (F44.7), and persistent postural-perceptual dizziness (PPPD, ICD-11 (World Health Organization (WHO), 2021) code AB32). Patients could present with several symptom types and diagnosis of mixed FND (F44.7) was given when motor symptoms (F44.4), functional seizures (F44.5), and sensory symptoms (F44.6) were present. Symptom severity was evaluated with the severity scale of the Clinical Global Impression (CGI (Busner and Targum, 2007)), as 0 = no symptoms; 1 = normal, not at all ill; 2 = borderline mentally ill; 3 = mildly ill; 4 = moderately ill; 5 = markedly ill; 6 = severely ill; 7 = among the most extremely ill patients. The CGI rating was based upon observed and reported symptoms during the study visit (approximately 2.5 h). The CGI was initially assessed by SW, JB and GV and then discussed with SA. Duration of symptoms was calculated in months from the beginning of first symptoms to the date of study inclusion. Psychotropic medication was recorded (i.e., benzodiazepines, antidepressants, neuroleptics, antiepileptics) and dichotomized into intake = yes/no for subsequent analysis. All subjects completed the Beck’s Depression Inventory (BDI (Beck, 1961)), State-Trait Anxiety Inventory (STAI (Spielberger et al., 1983)) and the Childhood Trauma Questionnaire (Bernstein et al., 2003). A total CTQ score was calculated as the sum of its 5 subscales (i.e., emotional neglect, emotional abuse, physical neglect, physical abuse, sexual abuse).

### 2.3 Stress biomarkers

To assess stress objectively salivary samples were collected for the analyses of cortisol and alpha-amylase (Salimetrics, High Sensitivity Salivary Cortisol EIA kit 1-3002 and Salivary a-amylase kinetic enzyme assay kit 1-1902). The detailed protocol for salivary cortisol collection can be found in (Weber et al., 2022). We calculated an estimate of the cortisol awakening response (CAR, i.e., the rapid increase in cortisol in the morning (Hellhammer et al., 2009, Wust et al., 2000)) using the area-under-the-curve with respect to ground (Pruessner et al., 2003, Stalder et al., 2016). In comparison to cortisol, which represents a slow response to hypothalamus–pituitary–adrenal (HPA)-axis activity, alpha-amylase can be used as an indicator for the rapid sympathetic – adrenal medullary response and has previously been found to be elevated in FND patients (Apazoglou et al., 2017). Saliva samples for analysis of alpha-amylase were collected before entering the MRI scanner using Salivette collection devices (Sarstedt, Germany).

### 2.4 Neuroimaging acquisition and pre-processing

Resting-state functional and structural MRI data were recorded using a 3 Tesla Scanner (Magnetom Prisma, Siemens, Germany). To avoid excessive head motion, we stabilized participants' heads using foam pads. Subjects were instructed to lay as calm as possible, to not fall asleep and to not think of anything. During the resting-state scan, participants were asked to fixate a white cross on a black background. For anatomical imaging, a sagittal-oriented T1-weighted 3D-MPRAGE sequence (TR = 2330 ms, TE = 3.03 ms, TI = 1100 ms, matrix 256 × 256, FOV 256 mm × 256 mm, flip angle 8°, resolution 1 mm^3^ isotropic, TA = 5:27 min) was acquired (Gallichan et al., 2016). Functional imaging data were acquired using a whole-brain interleaved multi-slice BOLD echo-planar-imaging (EPI) sequence (TR = 1300 ms, TE = 37 ms, flip angle = 52°, FOV = 230 mm, voxel size = 2.2 mm^3^ isotropic, TA = 6:39 min, for a total of 300 functional volumes). Imaging data were pre-processed using SPM12 (https://www.fil.ion.ucl.ac.uk/spm/software/spm12/) in MATLAB (R2017b, MathWork Inc., Natick, USA). Functional volumes were first realigned and co-registered to the structural T1 volume. They were then detrended and covariates of no interest were regressed out (including constant, linear, and quadratic trends, average white matter/cerebrospinal fluid time courses, translational and rotational motion time courses upon realignment, and global signal). Functional data were then filtered using a high-pass filter at 0.01 Hz. Lastly, functional volumes were warped into MNI standard space and smoothed using a spatial Gaussian kernel of 5 mm full width at half maximum.

Moreover, as head motion is known to affect FC analyses such that FC in large-scale distributed networks decreases, while local FC increases (Van Dijk et al., 2012), functional images were checked for excessive translation and rotation with the framewise displacement (FD) criterion of (Power et al., 2014) at a threshold of FD > 0.5 mm. Subjects in which more than 50 % (i.e., > 150) of volumes showed too high motion were excluded from further analysis.

### 2.5 Resting-state functional dynamics

To characterize large-scale brain network dynamics at rest in patients with FND and HC, we applied a two-step data-driven methodological approach. First, we computed *static* functional connectivity between 17 resting-state networks. In a second step, we examined *dynamic* network alterations in the most discriminative networks using a co-activation pattern (CAP) analysis (Bolton et al., 2020, Liu et al., 2018, Liu et al., 2013, Liu and Duyn, 2013). On a conceptual level, CAP analysis works at a single-volume temporal resolution and thus deviates from the conventional methods applied to the temporal domain (Liu and Duyn, 2013, Liu et al., 2013). CAP analysis allows to identify transient co-(de)activation patterns or brain “states” that reappear throughout a resting-state fMRI scan (Liu et al., 2018).

#### 2.5.1 Resting-state functional network connectivity

**Fig. 1:**
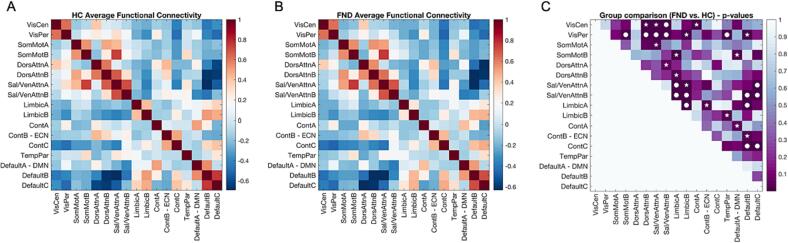
Resting-state network connectivity in healthy controls (HC) and FND patients. Average FC values within and between resting state networks (RSN) in A) HC and B) FND patients; C) p-values for the differences between FND patients and HC using multiple t-tests (two-sided) corrected for multiple comparison using FDR. RSN labels follow the convention of Yeo (Yeo et al., 2011). Significance code: *P < 0.01 (non-corrected); ●P surviving FDR-correction (P < 0.05). Abbreviations: Cont = Executive control, Default = Default mode DorsAttn = Dorsal attention, FC = Functional Connectivity, Sal/VenAttn = Salience/Ventral attention, SomMot = somatomotor, TempPar = Temporoparietal, VisCen = Central vision, VisPer = Peripheral Visual, RSN = Resting-State Networks.

To assess the resting-state FC of the brain, we first parcellated each participant’s functional brain data into 17 resting-state networks (RSN) according to the convention of (Yeo et al., 2011). The Yeo atlas comprises diverse RSN such as the default mode network (DMN), dorsal and ventral attention networks, limbic network, somatomotor network, as well as executive control networks complying with functional topography and is therefore optimal to study functional network organisation. Then, for each participant an individual structural brain atlas was built using a customized version of the IBASPM toolbox (Aléman-Gomez et al., 2006). The individual structural atlas was normalized to standard space and mapped onto the native resolution of the functional data, so that each voxel of the functional data was assigned to one of the networks. The network-averaged time courses were extracted, and functional network connectivity was computed using Pearson’s correlation coefficient between the time series of each of the networks, producing an individual 17 × 17 FC matrix for each subject, Fig. 1. The correlation coefficients were further *z*-scored using Fisher *z* transformation. Significant differences in functional network connectivity between patients and controls were assessed using two-tailed multiple *t*-tests, corrected using false discovery rate (FDR) at a significance threshold of *P* < *α,* where the *α*-level was set to 0.05.

#### 2.5.2 Insular and amygdalar co-activation patterns

The *static* functional connectivity analysis revealed the salience network (SN) and limbic network to be differently connected in patients as compared to HC (see 3. Results). Thus, we selected the insula and the amygdala as two key regions being represented within these networks and as they are frequently reported to be altered in FND (Demartini et al., 2021, Drane et al., 2020, Pick et al., 2019), to analyse their spatial and temporal dynamics, using a CAP analysis (Bolton et al., 2020, Liu et al., 2018, Liu et al., 2013, Liu and Duyn, 2013). The seeds were defined using the automatic anatomic labelling (AAL) atlas (Tzourio-Mazoyer et al., 2002) to create a bilateral insula and amygdala mask, respectively. The analysis was restricted to voxels within the grey matter. The seed time series were scrubbed at 0.5 mm, extracted, and *z*-scored in time. To identify those time-points corresponding to high-amplitude events (activation) within our seeds, we thresholded the time-series at 0.84 SD which corresponds to the 80th percentile, representing high-amplitude blood oxygenation level-dependent signals (Liu and Duyn, 2013, Liu et al., 2013); i.e., volumes in which the seed region was highly activated. To generate CAPs, we used a customized version of the *TbCAPs* toolbox (https://github.com/FND-ResearchGroup/PCA_CAP_SW), which is described in detail in (Bolton et al., 2020). To identify the optimal number of clusters *K*, a consensus clustering approach was performed using the frames from HC, which is advised when patterns of activity are assumed to differ in diseased subjects (Bolton et al., 2020, Monti et al., 2003). Due to the high dimensionality of the data, and the consequential high computational load for clustering approaches, an additional principal component analysis (PCA) step was introduced to reduce the dimensionality of the data. Hence, we concatenated the data of each subject (of size *n* × *t*, where *n* is the number of grey matter voxels and *t* the number of timepoints selected per subject) into a data matrix *X* with dimensionality *n* × *T*, where *T* is the selected number of timepoints across all subjects and *T*≪*n*. The *X* matrix was then centred by subtracting the mean of each voxel. *X* further served as an input to PCA. The PCA projected data (scores) *W* (of dimension *T* × *T*) were used as input for consensus clustering. Based on the output of the consensus clusters, the cumulative distribution of consensus values was computed to further calculate the proportion of ambiguously clustered pairs (PAC) to evaluate the stability of the individual cluster sizes (Șenbabaoğlu et al., 2014). The stability measure, defined as *1-PAC*, and associate consensus matrices, can be found in Supplementary Figs. 1, 2 and 4, 5 (for insular and amygdalar CAPs, respectively). Based on the stability measure and the consensus matrices, the dimensionality-reduced selected fMRI volumes were clustered into four (using the insula as seed) or three (using the amygdala) different states (CAPs) using the *k*-means algorithm. The individual CAPs were then reconstructed back by multiplying the PC scores with the transposed eigenvectors and adding back the mean. The CAPs were subsequently spatially *z*-scored, representing the distinct insular and amygdalar CAPs with positive and negative contributions (Liu and Duyn, 2013, Liu et al., 2013). Lastly, each individual frame of the FND patients was then assigned to its most similar CAP by a matching process, in which the largest spatial correlation between an FND frame and the CAPs was compared to the distribution of spatial correlations of the frames from HC that belong to the best matching CAP. When the 5th percentile of this distribution was exceeded, assignment was performed. See Supplementary Material for more details.

To characterize the temporal properties of the obtained CAPs, we calculated the average duration of a CAP (average number of consecutive volumes assigned to one CAP multiplied by the TR), the number of entries (how many times a subject transitioned to a specific CAP), the number of volumes corresponding to a CAP (volumes assigned to a CAP), and relative temporal occurrence (defined as the number of volumes assigned to one CAP normalized by the number of selected volumes). The analyses were repeated with the second most stable cluster number (Supplementary Figs. 3 and 6). The number of excluded fMRI frames (derived from FD), age, sex, psychotropic medication (dichotomous), depression (BDI) and anxiety (STAI) scores were added as covariates.

In summary, apart from the spatial information, this method allows to capture the temporal characteristics of the dynamic brain such as duration or occurrence of individual brains states. Therefore, this method allows to better characterize the dynamic functional alterations of brain networks in FND patients.

### 2.6 Relationship between CAPs, stress biomarkers, and clinical scores

As temporal characteristics of brain states were found to be a representative biomarker for neuropsychiatric disorders (Khanna et al., 2014, Michel and Koenig, 2018), we explored the multivariate patterns of correlation between those CAPs that were altered in FND compared to HC (i.e., CAP2_Ins_, CAP4_Ins_), stress biomarkers (i.e., CAR, alpha-amylase), and clinical scores (i.e., symptoms durations, clinical global impression score and total CTQ score) in FND patients using a partial least squares correlation analysis (PLSC (Krishnan et al., 2011, McIntosh and Lobaugh, 2004)). We implemented our analysis using the publicly available PLS toolbox (https://github.com/FND-Research Group/myPLS_SL.git) (Loukas et al., 2021, Zöller et al., 2019). PLSC calculates correlation weights by detecting linear combinations of CAPs temporal characteristics and stress biomarkers/clinical scores such that their covariance is maximized across subjects.

## 3 Results

### 3.1 Clinical and demographic characteristics

In total, eight patients had mixed symptoms (F44.7), in 30 patients at least two symptom categories were present, and 48 patients had only one type of symptom category, Table 1. Levels of diagnostic certainty for functional seizure patients were: seven probable, three clinically established, and four documented, according to diagnostic criteria of (Lafrance et al., 2013). Our cohort represents a rather chronic patient population with an average duration of illness of 6.25 years and patients’ severity ranged from “normal, not at all ill” to “moderately ill”; i.e., representing a rather mild to moderate disease severity overall. Data from one HC and five FND patients had to be excluded due to too high motion artefacts, one patient due to a bleeding cyst, and one patient due to drug abuse. One HC did not finish the resting-state acquisition in the MRI. This leads to a total sample size of 79 FND patients and 74 HC. After exclusion of subjects, the two groups still differed in terms of number of discarded volumes (FND 5.68 % versus HC 1.61 %, *Z* = −5.1, *P* < 0.001) but not in terms of FD. Groups did not differ in the number of selected volumes for the analyses. Patients had significantly higher depression and anxiety scores, reported on more emotional and physical trauma, exhibited lower CAR, but did not differ in their alpha-amylase levels, Table 1.

**Table 1:** Demographic, behavioural, and clinical data.

		FND (N = 86)	HC (N = 76)	Statistics
Age, mean (SD), years, [range]		37.7 (14.2), [17–77]	33.1 (10.9), [18–62]	*Z* = −1.86, *P* = 0.06
Sex (females/males)		64/22	55/21	*X^2^*(1, 162) = 0.01, *P* = 0.9
Disease severity (CGI, median, quantile)		2 [1–4]	*NA*	
Duration of illness (in months)		75 (166)		
Symptom subtypes according to (Hallett et al., 2022)^a^			*NA*	
	Functional movement disorder	45 sensorimotor 25 gait disorder 17 tremor 12 myoclonus 8 dystonia 5 speech disorder		
	Functional seizures	15		
	Persistent perceptual-postural dizziness (PPPD)	7		
	Functional cognitive disorder	0		
ICD-10/11 classification		63 F44.4 7 F44.5 30 F44.6 8 F44.7 7 PPPD	*NA*	
Psychotropic medication		14 benzodiazepines 29 antidepressants 6 neuroleptics 9 antiepileptics 6 opioids	0/76	
**BDI score, mean (SD)**		**14.4 (9.96)**	**4.59 (6.28)**	***Z* = −7.61, *P* < 0.0001**
**STAI-S score, mean (SD)**		**37.2 (10.9)**	**32.1 (7.67)**	***t(*156.68) = 3.22, *P* = 0.002**
**STAI-T score, mean (SD)**		**45.5 (13.0)**	**33.9 (7.11)**	***t*(135.07) = 7.14, *P* < 0.001**
Alpha-amylase [U/ml], mean (SD)		184.4 (182.1)	140.1 (97.7)	*Z* = −1.29, *P* = 0.2
CAR (AUCg)^b^ [ng/ml], mean (SD)		257.32 (120.4)	306.23(131.0)	*F*(138,1) = 5.8, *P* = 0.01
**CTQ total score, mean (SD)**		**43.2 (17.0)**	**36.3 (13.9)**	*Z* = −3.11, *P* = 0.002 **
	**Emotional Abuse, mean (SD)**	**10.1 (5.14)**	**8.16 (4.17)**	*Z* = −3.13, *P* = 0.002 *
	**Emotional Neglect, mean (SD)**	**11.1 (5.12)**	**8.80 (4.15)**	*Z* = −2.58, *P* = 0.01*
	**Physical Abuse, mean (SD)**	**7.28 (4.03)**	**5.87 (1.98)**	*Z* = −2.34, *P* = 0.02 *
	**Physical Neglect, mean (SD)**	**7.71 (3.05)**	**6.79 (2.83)**	*Z* = −2.31, *P* = 0.02 *
	Sexual Abuse, mean (SD)	6.96 (3.89)	6.72 (3.95)	*Z* = −0.89, *P* = 0.37

Significance code: ***P < 0.001, **P < 0.01, *P < 0.05. Abbreviations: BDI: AUCg: Area-under-the-curve with respect to ground, Beck’s Depression Inventory, CAR: Cortisol awakening response, CGI: Clinical Global Impression Score, CTQ: Childhood Trauma Questionnaire, FND: functional neurological disorder, HC: healthy controls, STAI: State-Trait Anxiety Inventory, SD: standard deviation, ns: not significant, NA: not applicable.

a Patients can present with several symptom types.

b CAR was corrected for age, gender, BDI, STAI-S, psychotropic medication, menstrual cycle and hormonal contraception.

### 3.2 Aberrant resting-state network connectivity in FND

Compared to HC, FC in FND was significantly increased between 1) the salience network (SN) and the limbic network (*P*_FDR_ < 0.003), 2) the SN and the default mode network (DMN) (*P*_FDR_ < 0.004) and 3) the executive control network (ECN) and the DMN (*P*_FDR_ < 0.005). Furthermore, FC was significantly decreased in FND between 1) the limbic network and the DMN (*P*_FDR_ = 0.003), 2) the central visual network and the SN (*P*_FDR_ = 0.0007), 3) the peripheral visual network and (a) the somatomotor network, (b) the dorsal attention network, (c) the SN and (d) the temporoparietal network (*P*_FDR_ < 0.0002), Fig. 1.

### 3.3 Distinct dynamic brain networks in FND

#### 3.3.1 Insular co-activation patterns

**Fig. 2:**
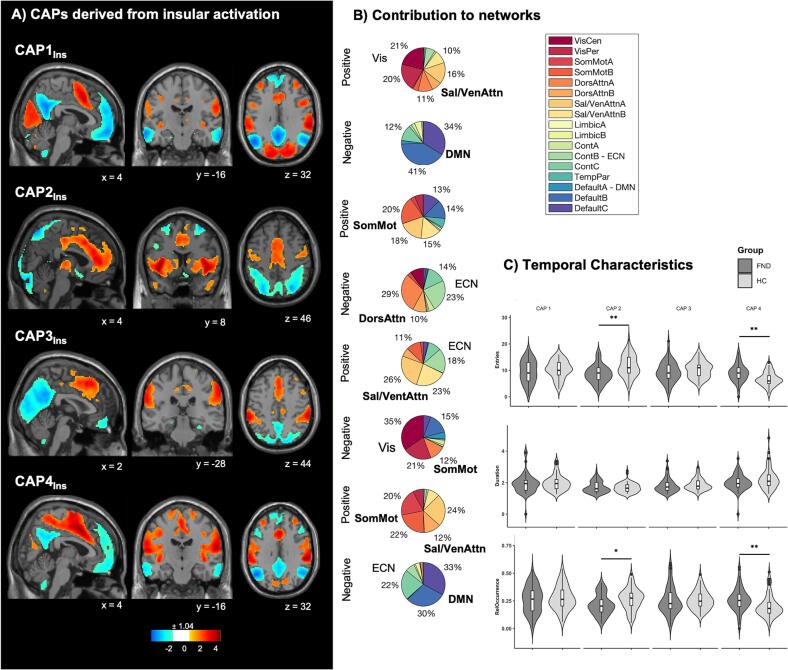
Co-activation pattern (CAP) maps based on insular seed activation. (A) Four CAPs were detected. CAPs were z-scored and only the 15 % most positive and 15 % most negative contributions are represented in colour (z = ± 1.04), with red representing positive contributions and blue negative contributions. Locations are displayed in Montreal Neurological Institute (MNI) standard space coordinates. (B) Pie charts illustrating the percentage of positive and negative contributions within the 17 RSN according to the convention of (Yeo et al., 2011). Seed voxels have been removed. (C) Temporal measures with numbers of entries (top), duration (middle) and relative occurrence (below) in FND patients and healthy controls. Violin plots visualize the distribution of the data. Asterisks indicate statistical significance for data adjusted for covariates (i.e., number of excluded fMRI frames [derived from FD], selected volumes, age, sex, psychotropic medication [dichotomous], depression [BDI] and anxiety [STAI] scores) and corrected for multiple comparisons with *P < 0.05, **P < 0.01. Boxplots: horizontal lines represent group median; box represents interquartile range and vertical line represents 1.5-times interquartile range. Abbreviations: BDI = Beck’s Depression Inventory, Ins = Insula, Cont = Executive control, Default = Default mode DorsAttn = Dorsal attention, Sal/VenAttn = Salience/Ventral attention, SomMot = somatomotor, TempPar = Temporoparietal, VisCen = Central vision, VisPer = Peripheral Visual, RelOccurrence = Relative Occurrence, RSN = Resting-State Network, STAI = State-Trait Anxiety Inventory. (For interpretation of the references to colour in this figure legend, the reader is referred to the web version of this article.)

We identified four insular CAPs. The first CAP (CAP1_Ins_) represents an insular activation pattern with co-activation of the visual network and SN and regions including the supplementary motor area (SMA); and a co-deactivation with the default mode network (DMN), and regions including the hippocampus and the cerebellum. The second CAP (CAP2_Ins_) exhibited an insular activation pattern with co-activation of the somatomotor network and the SN, and regions including the caudate and the thalamus; and a co-deactivation with the executive control network (ECN) and the dorsal attention network, as well as the olfactory tubercle, parahippocampal regions and the cerebellum. The third CAP (CAP3_Ins_) denotes an insular activation pattern with co-activation of the SN and ECN, including the middle cingulate cortex (MCC) and the supramarginal gyrus; as well as co-deactivation of the visual network. The last CAP (CAP4_Ins_) denotes an insular activation pattern with co-activation of the somatomotor network and SN, including the SMA, the MCC and the precentral gyrus; and a co-deactivation with the DMN and ECN, including the posterior cingulate cortex (PCC), Fig. 2. Most functional volumes were assigned to CAP1_Ins_ (27.93 %). 26.48 % of the volumes were assigned to CAP2_Ins_, 25.59 % to CAP3_Ins_, and 20 % to CAP4_Ins_, respectively. Adjusted for covariates of no-interest, patients were found to entered CAP2_Ins_ less frequently (*P_FDR_* = 0.00002) and entered CAP4_Ins_ more often than HC (*P_FDR_* = 0.00002). Correspondingly, CAP2_Ins_ had a lower relative occurrence (*P_FDR_* = 0.01) and CAP4_Ins_ had a higher relative occurrence (*P_FDR_* = 0.006) compared to HC. Further, patients had a shorter duration of CAP4_Ins_ compared to HC (*P* = 0.018), which did not survive FDR correction (*P_FDR_* = 0.07), Fig. 2C.

#### 3.3.2 Amygdalar co-activation patterns

**Fig. 3:**
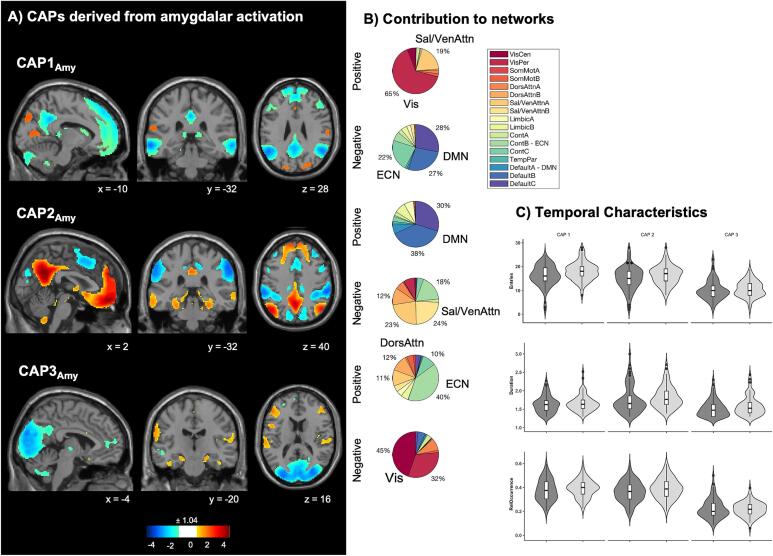
Co-activation pattern (CAP) maps based on amygdalar seed activation. (A) Three CAPs were detected. CAPs were z-scored and only the 15 % most positive and 15 % most negative contributions are represented in colour (z = ± 1.04), with red representing positive contributions and blue negative contributions. Locations are displayed in Montreal Neurological Institute (MNI) standard space coordinates. (B) Pie charts illustrating the percentage of positive and negative contributions within the 17 RSN according to the convention of (Yeo et al., 2011). Seed voxels have been removed. (C) Temporal measures with numbers of entries (top), duration (middle) and relative occurrence (below) in FND patients and healthy controls. Violin plots visualize the distribution of the data. Boxplots: horizontal lines represent group median; box represents interquartile range and vertical line represents 1.5-times interquartile range. Abbreviations: Amy = Amygdala, BDI = Beck’s Depression Inventory, Cont = Executive control, Default = Default mode DorsAttn = Dorsal attention, Sal/VenAttn = Salience/Ventral attention, SomMot = somatomotor, TempPar = Temporoparietal, VisCen = Central vision, VisPer = Peripheral Visual, RelOccurrence = Relative Occurrence, RSN = Resting-State Network, STAI = State-Trait Anxiety Inventory. (For interpretation of the references to colour in this figure legend, the reader is referred to the web version of this article.)

Three amygdalar CAPs were identified. The first CAP (CAP1_Amy_) demonstrated an amygdalar activation pattern with co-activation of the visual network and the SN; and a co-deactivation with the DMN and the ECN. The second CAP (CAP2_Amy_) characterizes an amygdalar activation pattern with co-activation of the DMN; and a co-deactivation of the SN and the ECN. The third CAP (CAP3_Amy_) represents an amygdalar activation pattern with co-activation of the ECN; as well as co-deactivation of the visual network, Fig. 3. 39.13 % of the selected volumes were assigned to CAP1_Amy_, 39.03 % of the volumes were assigned to CAP2_Amy_, and 21.84 % to CAP3_Amy_, respectively. Without adjusting for covariates, but FDR-corrected, patients were found to enter CAP1_Amy_ less frequently than HC (*t*_CAP1Amy_(142) = −2.68, *P* = 0.024). Results did not survive adjusting for covariates, Fig. 3C.

### 3.4 Association with clinical scores and stress biomarkers

**Fig. 4:**
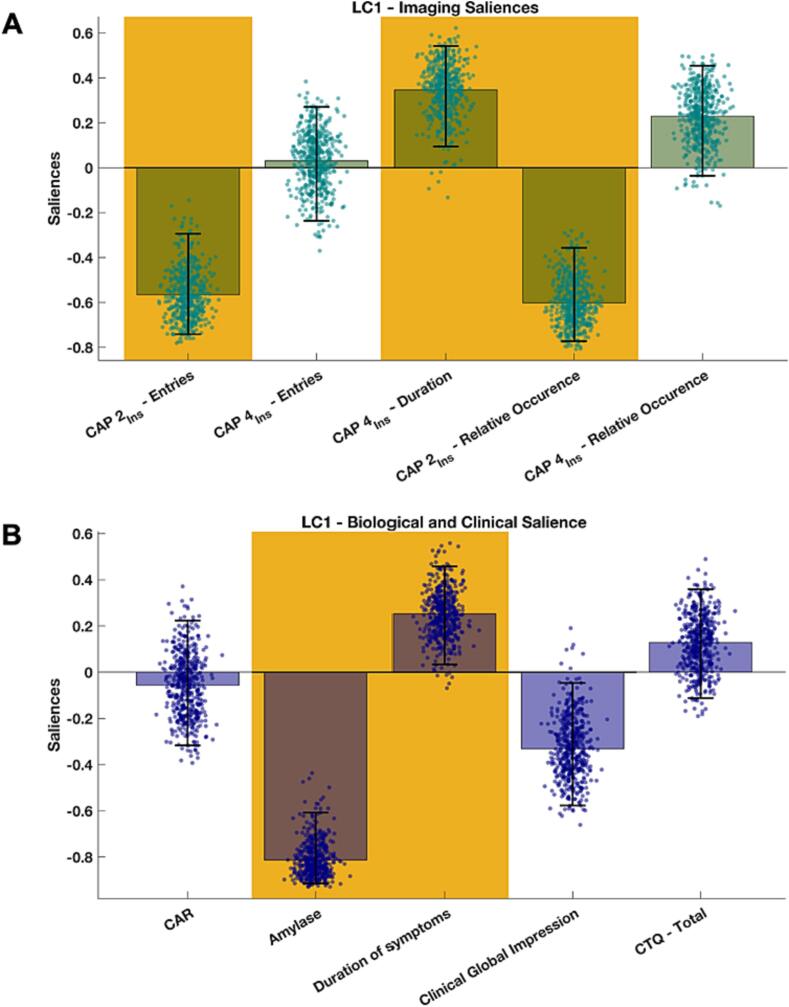
Partial least squares correlation (PLSC) results of the CAPs temporal metrics in FND patients. Salience weights of the significant PLSC component (P = 0.029) are presented for stress biomarkers (corrected for age, gender, menstrual cycle, hormonal contraception, menopause, and smoking) and clinical scores (A), and for imaging features (corrected for the number of excluded fMRI frames, the number of selected volumes, age, sex, psychotropic medication, BDI, and STAI-S) (B). Accentuated salience weights indicate statistical significance and were robust (based on bootstrapping). Error bars represent the 5th to 95th percentiles of bootstrapping and accentuated bars show robustness. The height of a bar represents the salience’s weight to the multivariate correlation pattern (i.e., higher bar equals a stronger contribution) and can be interpreted analogously to correlation coefficients as the data were standardized (i.e., bars pointing in the same direction depict a positive correlation whereas bars pointing in different directions depict an inverse correlation). The permutation null distribution and the bootstrap mean percentiles are reported in Supplementary Fig. 7 and Supplementary Table 1. Abbreviations: CAP = Co-activation pattern, CAR = Cortisol Awakening Response, CTQ = Childhood Trauma Questionnaire, Ins = Insula.

One patient was excluded from this analysis due to missing data on the CAR. One PLSC component was statistically significant (*P* = 0.029). The saliences are shown in Fig. 4. A significant positive correlation was found between the CAP2_Ins_ – entries and relative occurrence, and CAP4_Ins_ – duration and relative occurrence with alpha-amylase and duration of symptoms – meaning that higher levels of alpha-amylase and a shorter duration of symptoms were conjointly associated with more entries and occurrences in CAP2_Ins_ and shorter duration and lower relative occurrence of CAP4_Ins_. The analyses were repeated in HC and only between stress biomarkers and clinical scores in Supplementary Fig. 8 and 9.

## 4 Discussion

Comparing a cohort of chronic FND patients with rather mild to moderate functional neurological symptoms to healthy controls, this study revealed temporal alterations in functional brain networks encompassing dynamic insular co-(de)activation with the salience, default mode, and somatomotor networks; as well as insular co-(de)activation with subcortical regions and wide-spread cortical networks. The temporal aberrancies in insular CAPs correlated with alpha-amylase and duration of symptoms.

### 4.1 Network disturbances in FND

In FND patients, FC was significantly higher between 1) the SN and limbic network, 2) the SN and the DMN, and 3) the ECN and the DMN. Functional abnormalities in the limbic network have recurrently been reported in FND and are thought to be involved in the pathophysiological mechanisms underlying symptoms (Aybek and Vuilleumier, 2016, Voon et al., 2016). Especially, enhanced amygdalar activity was frequently found in patients, and was linked to impaired motor behaviour (Aybek et al., 2015, Diez et al., 2019, Hassa et al., 2021, Voon et al., 2010), implying a direct limbic influence on motor control (Voon et al., 2010). At the circuit level, many of the limbic emotional processing functions overlap in the salience circuits, emphasizing their importance in interrelated network dysfunctions. Likewise, increased co-activation of the insula with the fronto-parietal network has been identified in functional seizures (van der Kruijs et al., 2014). Particularly the insula is involved in emotional processing and -awareness, as well as interoception (Uddin, 2017), and has previously been associated with a reduced interoceptive accuracy (Goodman et al., 2022, Koreki et al., 2020, Sojka et al., 2021), to alter motor execution by bypassing executive control modulated by the emotional state (van der Kruijs et al., 2012), and with increased self-monitoring in FND patients (Pareés et al., 2013).

### 4.2 Reduced coupling between the default mode and salience-somatomotor networks

Further evidence arises from our CAP analysis. Of particular interest are the increased temporal dynamics in patients regarding CAP4_Ins_, which reflect stronger anti-correlations, i.e., reduced insular coupling with the DMN and the somatomotor and salience networks. In particular, the insula as an integral hub of the SN, has been suggested to facilitate attention and reactivity towards salient stimuli and quickly access the motor system through its strong coupling with other networks (Menon and Uddin, 2010). Particularly, aberrant functional inter-network coupling of the DMN has been associated with enhanced self-referential processing (van Buuren et al., 2010), which can further affect orientation of attention (Zhao et al., 2018), for which in particular the coupling between the DMN and somatomotor network has been suggested to help regulating attentional processes in FND (Kozlowska et al., 2018). Likewise, in FND, increased self-referential processing and shifted attention towards self-relevant cues have been observed (Huys et al., 2021, Pareés et al., 2012).

Correspondingly, internal and external somatosensory stimuli are integrated within the DMN and SN (Barrett and Simmons, 2015, Kleckner et al., 2017, Paulus et al., 2019). Therefore, reduced coupling between the DMN and SN might be associated to dysfunctional bottom-up integration of somatosensory input, which could lead to a misinterpretation of the contextual information in higher-order regions (Fox et al., 2018). Aberrant somatosensory integration has been supported by findings from other resting-state studies in FND on enhanced functional propagation from motor-limbic information to the multimodal integration network (Diez et al., 2019). Similarly, patients were found to remain longer in brain states associated with attentional processes potentially associated to the misinterpretation of sensory information (Marapin et al., 2020). Such a misapprehension of sensory information has been suggested causing a misperception of somatic sensations, which could explain the appearance of functional symptoms (Beissner et al., 2015, Fiorio et al., 2022, Pareés et al., 2013, Pareés et al., 2012, Van den Bergh et al., 2017). In essence, aberrant insular inter-network coupling of the DMN and the salience and somatomotor networks might be implicated in the misdirected attention and impaired sensory integration which might interfere with the proper execution of motor functions observed in FND (Aybek et al., 2015, Aybek et al., 2014, Diez et al., 2019, Hassa et al., 2017).

### 4.3 Altered cortical-subcortical coupling in FND

The second interesting pattern encompasses CAP2_Ins_ and its related temporal measures. CAP2_Ins_ does not clearly overlap with one particular network, and rather shows insular co-(de)activation with various cortical networks and subcortical structures such as the thalamus or the caudate. Moreover, less entries were found into CAP2_Ins_ in patients reflecting lesser co-activations, i.e., reduced cortical-subcortical coupling. The insula has been suggested to play a major role in the flexible switch between large-scale networks (Menon and Uddin, 2010). Likewise, previous research suggested that certain brain states might facilitate the appearance of/or transition into other brain states (Michel and Koenig, 2018), and that changes in cortical brain states temporally coincide with subcortical shifts (Favaretto et al., 2022) suggesting that subcortical states may coordinate large-scale cortical network integration (Bell and Shine, 2016, Pessoa, 2014). Thus, these findings here might provide first insights into altered cortical network integration orchestrated through cortical-subcortical coupling in FND. However, as cortical-subcortical coupling has never been subject to a scientific study in FND, it will be of great interest to further investigate on these connectivity patterns.

### 4.4 Functional alterations and stress regulation

Further evidence comes from our PLSC analysis in which the temporal characteristics of CAP2_Ins_ and CAP4_Ins_ conjointly correlated with alpha-amylase and duration of symptoms. In other words, lower alpha-amylase levels and longer symptom duration correlated with lesser entries into CAP2_Ins_. Likewise, lower alpha-amylase levels and longer symptom duration correlated with a longer duration of CAP4_Ins_. The correlation with symptom duration is in line with the results found in the simple group-wise comparison of the temporal characteristics while the amylase levels are converse. These findings might suggest a maladaptive process in which chronification of symptoms could add to a long-term alteration in functional integration, whereas higher levels in alpha-amylase might reflect compensatory mechanism to stress-related changes in cortical and subcortical network connectivity in FND, underscoring its potential relevance of stress regulatory processes in functional alterations in FND. Previously, acute stress has been found to reduce inter- and intra-network coupling of the DMN and ECN (van Leeuwen et al., 2020), whereas it increased intra-network connectivity of the SN (van Leeuwen et al., 2020, Zhang et al., 2019). The SN has been suggested to play a pivotal role in the response to acute stress, whereas the DMN has rather been associated to post-stress homeostatic restoration and emotion regulation (van Oort et al., 2017). Likewise, reduced functional integration was found in anxiety disorders when anticipating stress potentially reflecting a failure to activate adaptive control processes (Cremers et al., 2015).

Providing a slightly different view on pathology, FND has previously been conceptualized as a disorder of altered functional organization in the brain resulting in a return into a more primitive brain state prioritizing homeostatic defensive functions (i.e., energy regulation) associated with rather reflexive and emotionally-valenced actions evolved to evade threat (Blakemore et al., 2016, Kozlowska et al., 2018). To that line, altered insular and amygdalar functional and structural connectivity patterns to higher-order cortical regions were found to be associated with early-life adversities in FND with a potential genetic involvement, pointing towards stress-related neuroplasticity priming the brain towards threat (Diez et al., 2020, Jungilligens et al., 2022). In order to maintain homeostasis, the brain continuously predicts the body’s energy needs through a process called allostasis (Sterling, 2012). Previous evidence supports the existence of an allostatic-interoceptive brain system – monitoring and regulating the body’s internal state – encompassing the DMN and SN, which strongly overlaps with CAP4_Ins_ (Kleckner et al., 2017). The association between alpha-amylase with temporal alterations in cortical-subcortical coupling, and somatomotor-SN to DMN coupling in FND could support the previous notion on functional alterations being associated with prioritizing allostatic mechanisms as a result to a state of chronic alert (elevated autonomic arousal) (Blakemore et al., 2016, Kozlowska et al., 2018). However, it will be of great interest to investigate whether these connectivity patterns are causally influenced through the sympathetic – adrenal medullary system. Based on these results herein it cannot be concluded whether these alterations might add to the development of FND or reflect a result of the disorder with the potential to be reverted.

### 4.5 Limitations

First, adopting a seed-based approach might be susceptible to noise. As CAPs analysis works at a single-volume temporal resolution, all selected timepoints show high activity in the seed region and consequently, so will the resulting CAPs (Liu et al., 2018). Therefore, co-activation with other regions might occur at chance-level. Furthermore, the selection of the insula and the amygdala as our seed regions might not optimally represent the salience- and limbic networks, as the anterior insula and the dorsal part of the amygdala are more directly associated with the salience network, while only the medial part of the amygdala is associated with the limbic network (Bickart et al., 2012). Selecting another seed, such as for example the anterior cingulate cortex, or using a unilateral mask could have led to different outcomes in this study. Second, the optimal cluster size has been selected based on consensus. Even though this is common practice, a different cluster size could have led to significantly different CAPs. Third, the initial PCA step might cause a loss of weaker networks, and conversely inflate the presence of dominant CAPs. Forth, there is no clear consensus on the physiological mechanisms underlying temporal alterations between groups. Thus, the results might have only little biological relevance regarding the pathophysiology of FND. Fifth, although our sample size is considerably large, the FND population is heterogenous in type and severity of symptoms, which impedes the generalizability of the results. It is also of note that patients present with a rather chronic FND and mild to moderate symptom severity and only few cases with severe neurological symptoms were present. Thus, the results might not generalize to (sub-)acute patients or those with very severe neurological symptoms. Additionally, patients often suffer from psychiatric comorbidities (Carson and Lehn, 2016), which is also reflected in our population. Even though our results remained significant upon correction for confounding effects, we did not perform a systematic psychiatric evaluation. Thus, we cannot exclude that these results are non-specific to FND. Furthermore, patients might differ in their predisposing vulnerabilities which could have affected the results. Sixth, alpha-amylase was collected directly before entering the scanner, which on one hand is appropriate to assess potential correlations between functional brain alterations and stress might also influence the results through nervosity – and as a result increased sympathetic activity – of participants upon entering the MRI scanner (*e.g.*, due to claustrophobia). Lastly, even though we corrected for psychotropic medication intake, the effect of patients’ medication intake on functional brain dynamics must be evaluated with caution.

## 5 Conclusion

Despite the large progress in neuroimaging research in FND within the last years, growing interest lies in investigating how neural correlates in FND relate to clinical characteristics or other objective biomarkers (Perez et al., 2021). Our study adds a new observation to current knowledge supporting *“Charcot’s dynamic lesion”* in the form of dynamic alterations in inter- and intra-network connectivity patterns in FND. We identified altered insular co-activation patterns with the SN, the somatomotor network, and co-deactivation patterns with the DMN, as well as an insular co-activation pattern with subcortical regions and wide-spread cortical networks. These alterations might be associated with attentional and interoceptive processes. In patients, temporal alterations could further be linked to alpha-amylase and duration of symptoms suggesting a potential influence of the stress system and chronification of symptoms on brain functional integration.

## Funding/support

This work was supported by the 10.13039/501100001711Swiss National Science Foundation (SNF Grant PP00P3_176985 for SA).

## CRediT authorship contribution statement

**Samantha Weber:** Conceptualization, Data curation, Formal analysis, Investigation, Methodology, Project administration, Software, Validation, Visualization, Writing – original draft, Writing – review & editing. **Janine Bühler:** Data curation, Project administration. **Serafeim Loukas:** Formal analysis, Methodology. **Thomas A.W. Bolton:** Methodology, Supervision, Writing – review & editing. **Giorgio Vanini:** Data curation. **Rupert Bruckmaier:** Funding acquisition, Resources. **Selma Aybek:** Conceptualization, Funding acquisition, Supervision, Resources.

## Declaration of competing interest

The authors declare that they have no known competing financial interests or personal relationships that could have appeared to influence the work reported in this paper.

## Data Availability

Data will be made available on request.
