# Supplementary material for: Transient resting-state salience-limbic co-activation patterns in functional neurological disorders
Source: Neuroimage Clin. 2024 Feb 28;41:103583. doi: 10.1016/j.nicl.2024.103583 (PMC10944183; doi:10.1016/j.nicl.2024.103583)
Supplement: Supplementary data 1 [file mmc1.pdf]

# Supplementary Material

## Transient resting-state salience-limbic co-activation patterns in functional neurological disorders

Authors: Samantha Weber[a,b,c,d], Janine Bühler[a,c], Serafeim Loukas[a,d,e], Thomas A.W. Bolton[f,g], Giorgio Vanini[a], Rupert Bruckmaier[h], Selma Aybek[a,i]

**Author affiliations:**

[a]Department of Neurology, Psychosomatic Medicine Unit, Inselspital Bern University Hospital, University of Bern, 3012 Bern, Switzerland

[b]University of Zurich, Psychiatric University Hospital Zurich, Department of Psychiatry, Psychotherapy and Psychosomatics, 8032 Zurich, Switzerland.

[c]Translational Imaging Center (TIC), Swiss Institute for Translational and Entrepreneurial Medicine, 3010 Bern, Switzerland.

[d]Institute of Bioengineering, Ecole Polytechnique Fédérale de Lausanne (EPFL), 1015 Lausanne, Switzerland

[e]Division of Development and Growth, Department of Pediatrics, University of Geneva, 1211 Geneva, Switzerland

[f]Department of Clinical Neurosciences, Neurosurgery Service and Gamma Knife Center, Centre Hospitalier Universitaire Vaudois, 1011 Lausanne, Switzerland

[g]Department of Radiology, Centre Hospitalier Universitaire Vaudois, 1011 Lausanne, Switzerland

[h]Veterinary Physiology, Vetsuisse Faculty, University of Bern, 3012 Bern, Switzerland

[i]Faculty of Science and Medicine, University of Fribourg, 1700 Fribourg, Switzerland

# CAPs Insula

**Supplementary Figure 1 Stability measure (1 – PAC).** To assess whether a certain cluster number is good, two given data points should consistently be clustered together or in different clusters across folds. The cumulative distribution of consensus values across all pairs of data points can be computed, which gives a quantification of the goodness of fit. We refer to this distribution as $P_k(c)\ with\ c \in [0,1]$. From this, the proportion of ambiguously clustered pairs (PAC) can be computed (Șenbabaoğlu et al., 2014) as $PAC_k = \sum_{C=C_T}^{1-C_T} P_k(c)$, with $c_T$ a threshold consensus value above which an assignment is judged as not sufficiently homogeneous across folds, and *k* the cluster number. A lower PAC thus represents a more robust cluster number. The stability measure is then derived as 1 – PAC, and therefore, greater values represent more robust clusters. The individual bars (coloured) reflect the different choices for the threshold $c_T$.

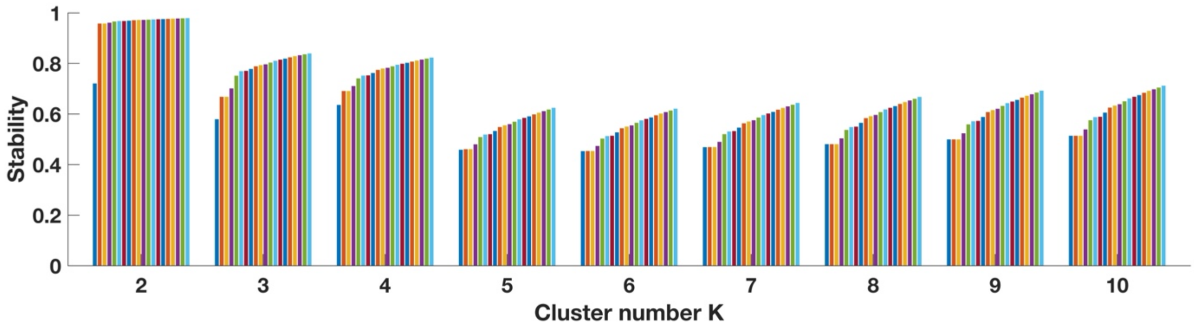

**Supplementary Figure 2 Consensus matrices.** The consensus matrices $C_k$ for a given number of clusters $k$, which summarize consensus values across all pairs of data points, are calculated by averaging, for each entry, over all folds where two data points jointly entered the computations. A cluster is considered stable, when two arbitrary data points are continuously clustered together, as represented by crisp boundaries in the consensus matrix.

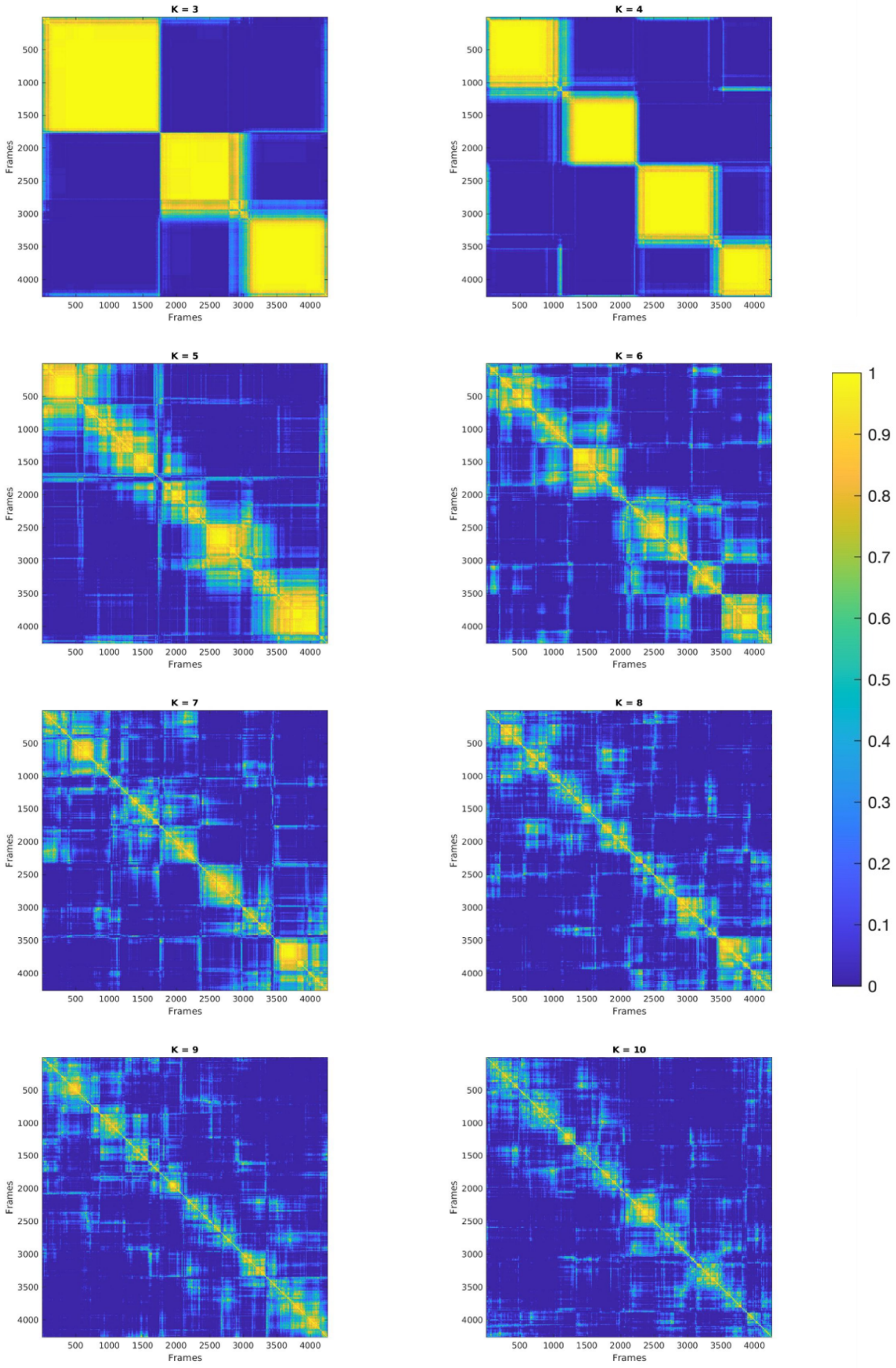

**Supplementary Figure 3 Co-activation patterns (CAPs) obtained for the second most stable cluster number (*K* = 3), based on insular seed activation.** Three CAPs were detected. CAPs were z-scored and only the 15% most positive and 15% most negative contributions are represented in colour ($z$ = ± 1.04), with red representing positive contributions and blue negative contributions. Locations are displayed in Montreal Neurological Institute (MNI) standard space coordinates. Abbreviations: Ins = Insula.

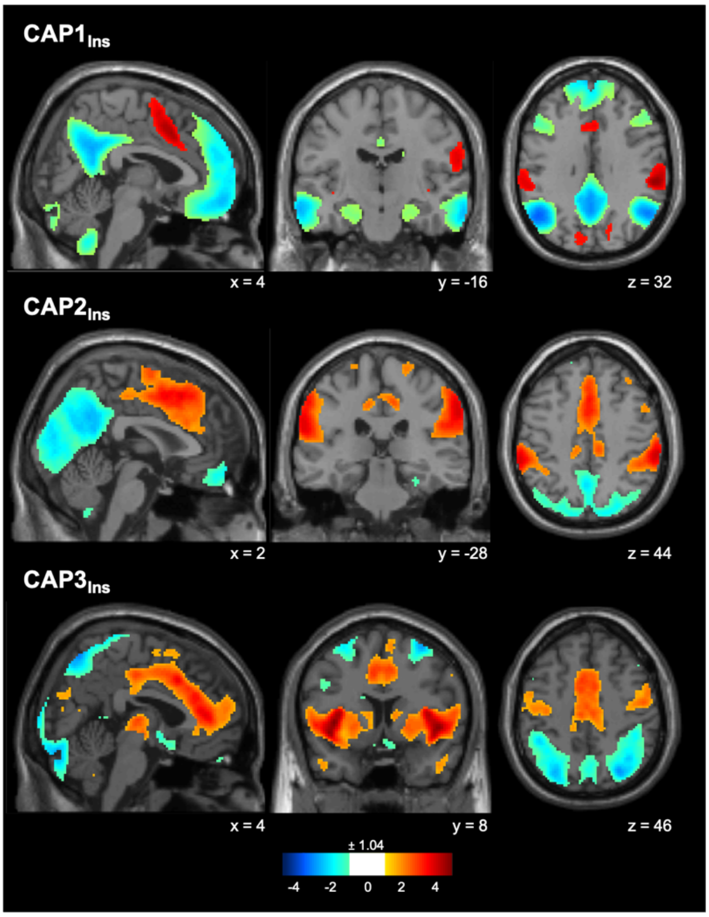

# CAPs Amygdala

**Supplementary Figure 4 Stability measure (1 – PAC).** To assess whether a certain cluster number is good, two given data points should consistently be clustered together or in different clusters across folds. The cumulative distribution of consensus values across all pairs of data points can be computed, which gives a quantification of the goodness of fit. We refer to this distribution as $P_k(c)\ with\ c \in [0,1]$. From this, the proportion of ambiguously clustered pairs (PAC) can be computed (Șenbabaoğlu et al., 2014) as $PAC_k = \sum_{C=C_T}^{1-C_T} P_k(c)$, with $c_T$ a threshold consensus value above which an assignment is judged as not sufficiently homogeneous across folds, and *k* the cluster number. A lower PAC thus represents a more robust cluster number. The stability measure is then derived as 1 – PAC, and therefore, greater values represent more robust clusters. The individual bars (coloured) reflect the different choices for the threshold $c_T$.

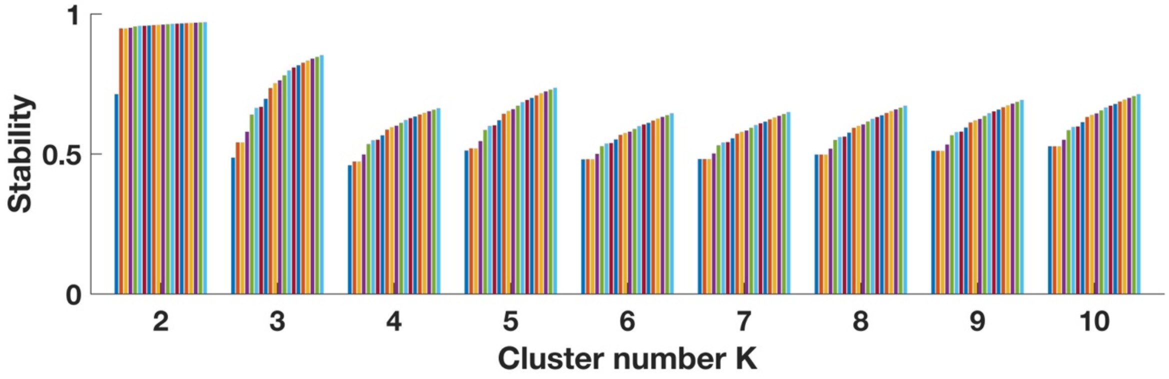

**Supplementary Figure 5 Consensus matrices.** The consensus matrices $C_k$ for a given number of clusters $k$, which summarize consensus values across all pairs of data points, are calculated by averaging, for each entry, over all folds where two data points jointly entered the computations. A cluster is considered stable, when two arbitrary data points are continuously clustered together, as represented by crisp boundaries in the consensus matrix.

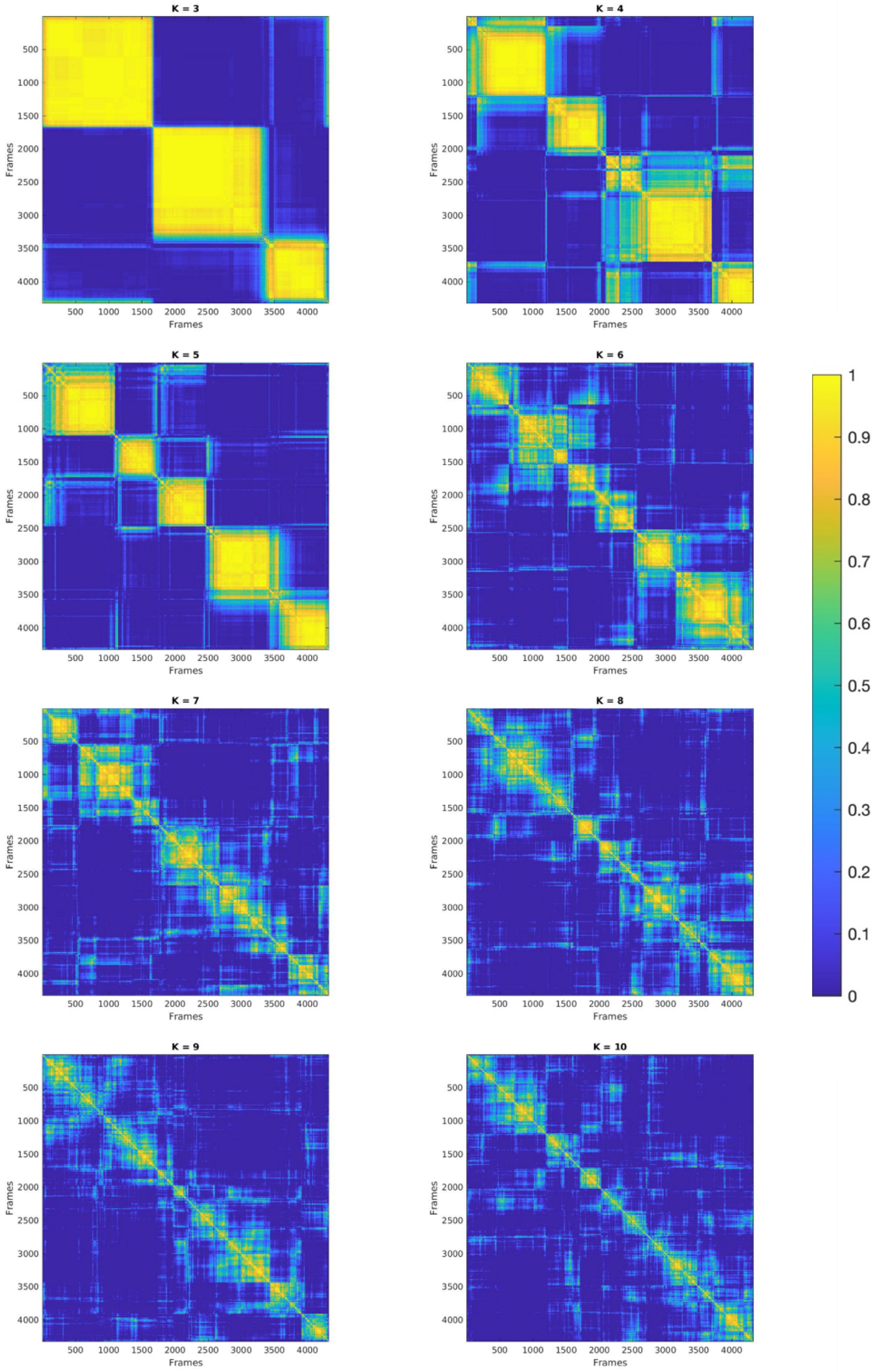

**Supplementary Figure 6 Co-activation patterns (CAPs) obtained for the second most stable cluster number (*K* = 5), based on amygdalar seed activation.** Five CAPs were detected. CAPs were *z*-scored and only the 15% most positive and 15% most negative contributions are represented in colour (z = ± 1.04), with red representing positive contributions and blue negative contributions. Locations are displayed in Montreal Neurological Institute (MNI) standard space coordinates. Abbreviations: Amy = Amygdala.

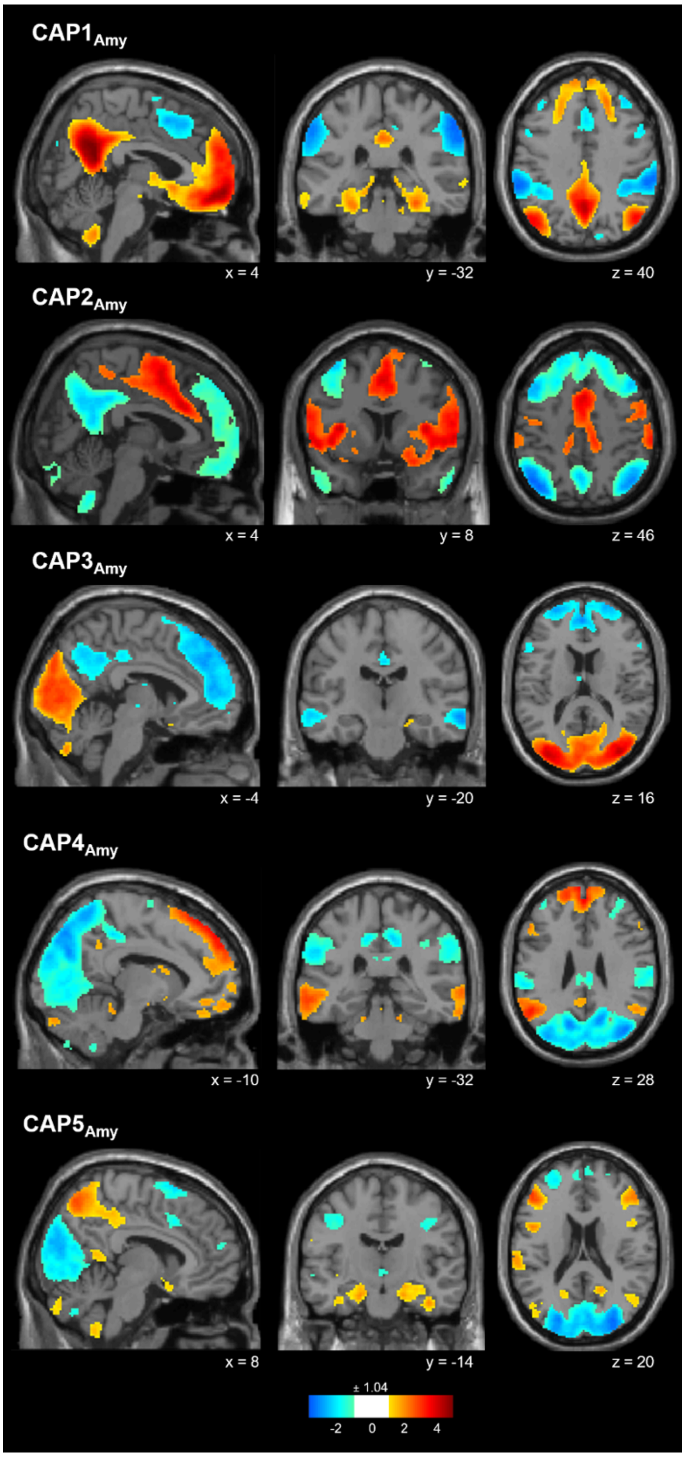

## Partial Least Squares Analysis

Data was standardized and a correlation matrix was calculated between CAPs temporal measures and stress biomarkers and clinical variables. To find individual weights of the corresponding data tables (CAPs measures x biomarkers and clinical scores), a single value decomposition (SVD) was used on the correlation matrix. The SVD leads to different correlation components consisting of a set of behavioural weights and outcome weights, indicating the strength of contribution of each weight to the multivariate pattern. The weights were used to calculate two sets of latent variables such that their covariance was maximized. Significance was evaluated by permutation testing (2000 permutations). Stability of the weights was assessed using bootstrapping (500 bootstrapping samples). PLSC allows for examining the relationship between multiple variables with different attributes. The PLSC analysis was conducted using the CAPs measures as design variables, and stress biomarkers and clinical scores as behavioural variables.

# Relationship CAPs temporal measures x biomarkers, clinical scores in FND

**Supplementary Figure 7: The permutation null distribution.** The histogram of the null distribution of the singular values is presented among with the observed (red line) singular value of the significant latent component (permutation testing, *P*=0.029). The y-axis represents frequency and the x-axis the singular values obtained by the permutation testing.

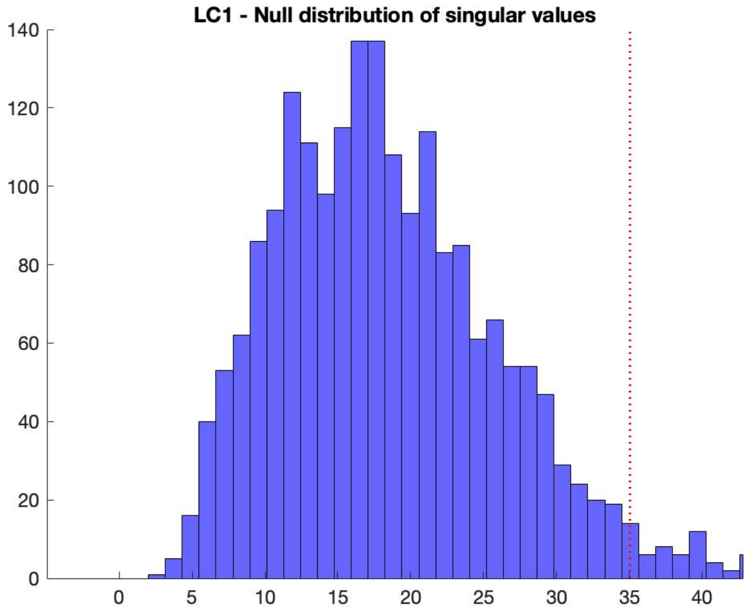


**Supplementary Table 1 Exact values of mean bootstrap weights and 5th to 95th percentiles for the identified statistically significant PLSC component (LC1, *P*=0.029). The significant rows are annotated in bold. Abbreviations: CI = confidence interval.**

| Mean bootstrapped imaging salience weights | Lower bound of CI | Upper bound of CI |
|---|---|---|
| **-0.6251637** | **-0.7419082** | **-0.2944002** |
| 0.05058795 | -0.2362332 | 0.27015822 |
| **0.35531427** | **0.09420084** | **0.54156923** |
| **-0.6504663** | **-0.7725843** | **-0.3564523** |
| 0.23928345 | -0.035867 | 0.45423718 |

| Mean bootstrapped behavioural salience weights | Lower bound of CI | Upper bound of CI |
|---|---|---|
| -0.063887 | -0.3170557 | 0.22398921 |
| **-0.8710538** | **-0.9141589** | **-0.6079267** |
| **0.27734731** | **0.03379079** | **0.45766406** |
| -0.3724484 | -0.5774922 | -0.0466712 |
| 0.14677981 | -0.1121197 | 0.35975375 |

## Relationship CAPs temporal measures x biomarkers, clinical scores in HC

To evaluate whether findings were specific to FND patients (with regard to HC), the multivariate relationship between aberrant CAPs temporal characteristics and behavioural characteristics was assessed in HC. Using stress biomarkers (CAR, alpha-amylase) as design variables and CAPs temporal characteristics as outcome variables, no significant multivariate correlation was identified in HC.

**Supplementary Figure 8: Explained covariance by each latent component.** None of the latent components (LC) reached significance.

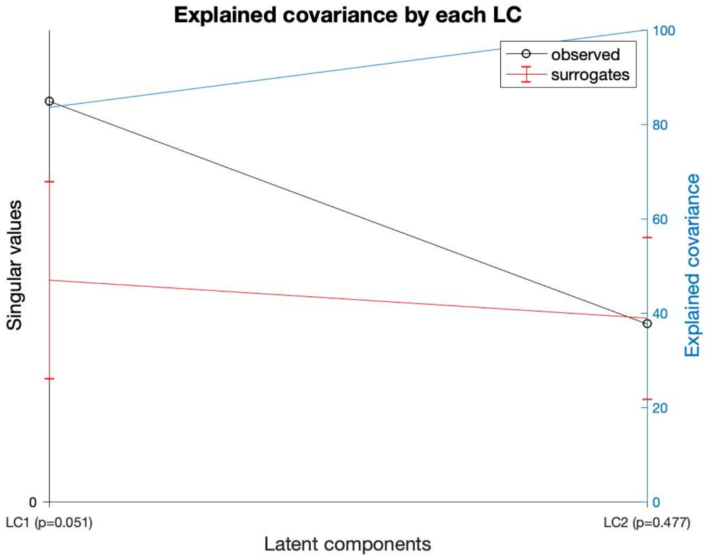

## Relationship between stress biomarkers and clinical scores in FND

No further correlations were found between stress biomarkers and clinical scores when calculating simple correlation coefficients. Within clinical scores, there was a significantly strong correlation between 1) depression (BDI) and trait anxiety (STAI-2) ($r$=0.82, *Pcorr*<0.0001), and 2) CGI and S-FMDRS (r=0.56, *Pcorr*<0.0001). A moderate significant correlation was found between 1) psychotropic medication intake and depression ($r$=0.56, *Pcorr* <0.0001), 2) psychotropic medication intake and trait anxiety ($r$=0.45, *Pcorr*=0.0002) and 3) depression and state anxiety ($r$=0.49, *Pcorr*<0.0001).

# Quality Control on CAP Methodology

The clustering was performed using the healthy controls as a reference population. A matching process was used to assign frames from FND patients to the HC-derived CAPs: the spatial correlation between a frame from FND patients and its closest (HC-derived) CAP was compared to the distribution of spatial correlations between the (HC) frames belonging to that CAP, and the CAP pattern itself. When the 5$^{th}$ percentile of this distribution was exceeded, the FND frame was assigned to the CAP at hand.

1. **Reference population: Whole population**

As quality control measure, we also report here the insular CAPs that were derived from using both groups together as input for the clustering process. Further, we calculated the exact voxel overlap in CAPs with those derived from using HCs as reference population. The CAPs derived from the whole population overall overlap anatomically with the CAPs reported in the main text which were derived only from HC data.

**Supplementary Figure 10: Co-activation pattern (CAP) maps based on insular seed activation using healthy controls and FND patients combined as reference population.** (**A**) Four CAPs were selected. CAPs were z-scored and only the 15% most positive and 15% most negative contributions are represented in colour (z = ± 1.04), with red representing positive contributions and blue negative contributions. Locations are displayed in Montreal Neurological Institute (MNI) standard space coordinates. (**B**) Pie charts illustrating the percentage of positive and negative contributions laying within the 17 RSN according to the convention of (Yeo et al., 2011). Seed voxels have been removed. Abbreviations: Ins = Insula, Cont = Executive control, Default = Default mode DorsAttn = Dorsal attention, Sal/VenAttn = Salience/Ventral attention, SomMot = somatomotor, TempPar = Temporoparietal, VisCen = Central vision, VisPer = Peripheral Visual, RSN = Resting-State Network.

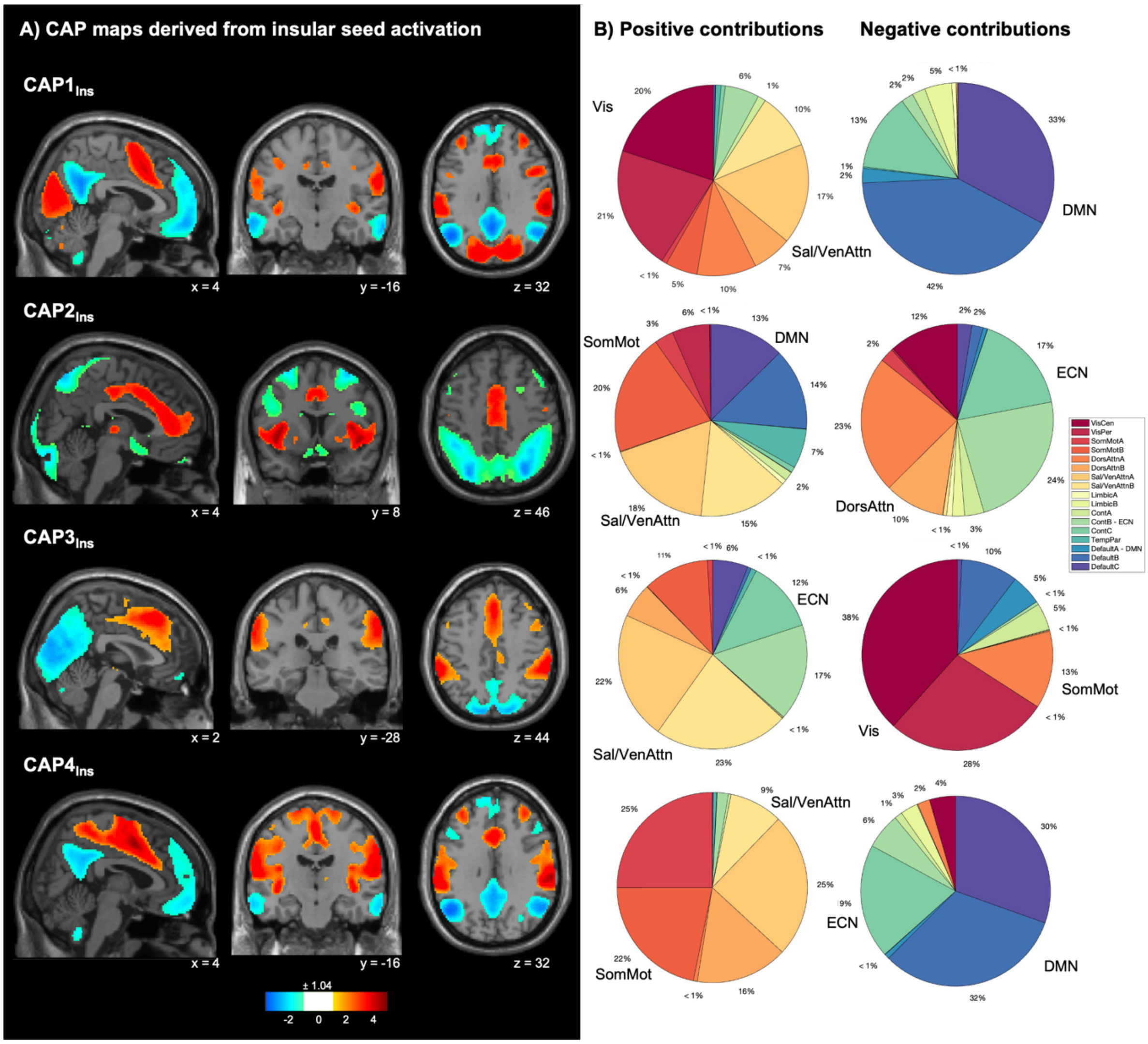
A) CAP maps derived from insular seed activation
CAP1Ins
CAP2Ins
CAP3Ins
CAP4Ins
x = 4
y = -16
z = 32
x = 4
y = 8
z = 46
x = 2
y = -28
z = 44
x = 4
y = -16
z = 32
± 1.04
-2 0 2 4
B) Positive contributions
Negative contributions
Vis
Sal/VenAttn
DMN
SomMot
DMN
ECN
Sal/VenAttn
DorsAttn
ECN
Sal/VenAttn
Vis
SomMot
Sal/VenAttn
SomMot
ECN
DMN
VisCen
VisPer
SomMotA
SomMotB
DorsAttnA
DorsAttnB
Sal/VenAttnA
Sal/VenAttnB
LimbicA
LimbicB
ContA
ContB - ECN
ContC
TempPar
DefaultA - DMN
DefaultB
DefaultC

## 2. Reference population: FND patients

As quality control measure, we also report here the insular CAPs that were derived from using the FND patients as input for the clustering process. Further, we calculated the exact voxel overlap in CAPs with those derived from using HC as reference population. The CAPs derived from the FND patients overall overlap anatomically with the CAPs reported in the main text which were derived from HC data only.

**Supplementary Figure 11: Co-activation pattern (CAP) maps based on insular seed activation using FND patients as reference population.** (**A**) Four CAPs were selected. CAPs were z-scored and only the 15% most positive and 15% most negative contributions are represented in colour (z = ± 1.04), with red representing positive contributions and blue negative contributions. Locations are displayed in Montreal Neurological Institute (MNI) standard space coordinates. (**B**) Pie charts illustrating the percentage of positive and negative contributions laying within the 17 RSN according to the convention of (Yeo et al., 2011). Seed voxels have been removed. Abbreviations: Ins = Insula, Cont = Executive control, Default = Default mode DorsAttn = Dorsal attention, Sal/VenAttn = Salience/Ventral attention, SomMot = somatomotor, TempPar = Temporoparietal, VisCen = Central vision, VisPer = Peripheral Visual, RSN = Resting-State Network.

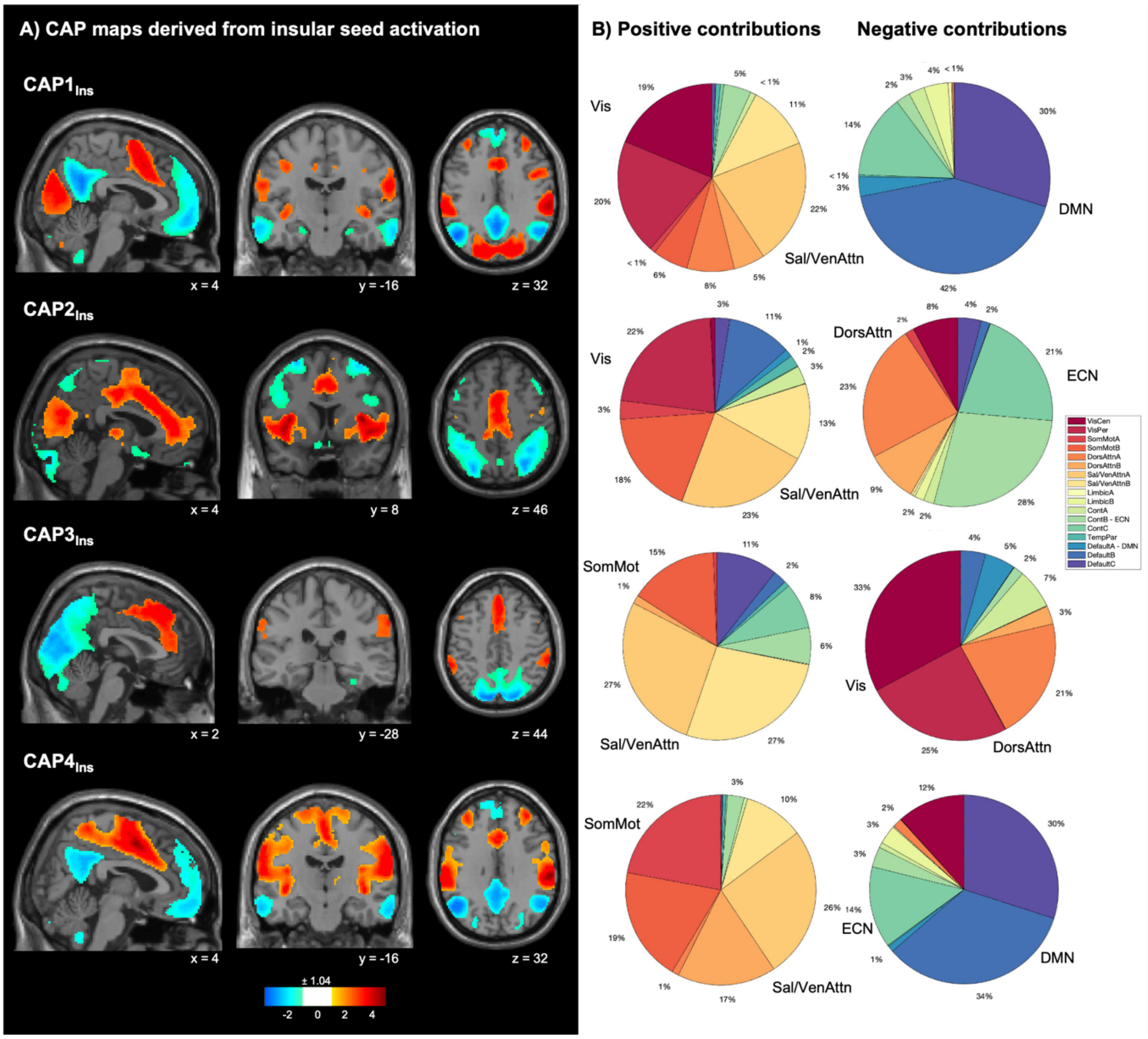
A) CAP maps derived from insular seed activation
CAP1Ins
x = 4
y = -16
z = 32
CAP2Ins
x = 4
y = 8
z = 46
CAP3Ins
x = 2
y = -28
z = 44
CAP4Ins
x = 4
y = -16
z = 32
± 1.04
-2
0
2
4
B) Positive contributions
Negative contributions
Vis
Sal/VenAttn
DMN
Vis
Sal/VenAttn
DorsAttn
ECN
SomMot
Sal/VenAttn
Vis
DorsAttn
SomMot
Sal/VenAttn
ECN
DMN
VisCen
VisPer
SomMotA
SomMotB
DorsAttnA
DorsAttnB
Sal/VenAttnA
Sal/VenAttnB
LimbicA
LimbicB
ContA
ContB - ECN
ContC
TempPar
DefaultA - DMN
DefaultB
DefaultC

## 3. Quality control initial PCA step

As the initial PCA step might cause a loss of weaker or less consistent networks, we compared the CAPs derived from 30 HC with and without the initial PCA step. Only 30 subjects were used due to the high computational load involved when using the whole dataset. Additionally, we compared the computational load with and without the PCA step, which was run on an Apple M2 MacBook Pro 2022 (macOS: Ventura 13.2.1, Memory: 16 GB), Supplementary Table 2. It must be considered that for this example, CAP analysis was run with only 30 subjects and for $K_{max}$ = 10 which significantly reduces the time as compared to using the full dataset and a higher $K$. Also, time taken for clustering does not increase linearly with a higher number of subjects, or a higher $K$ and rather increases exponentially.

**Supplementary Table 2 Computation load of co-activation pattern analysis on 30 subjects comparing with and without initial PCA step.**

| Step | Time taken without PCA | with PCA |
|---|---|---|
| Loading subjects | 12.1 min (24.2 sec per subject) | |
| Compute PCA | *not applicable* | 1.01 min |
| Consensus clustering ($K_{max}$ = 10, $P_{CC}$ = 80%, $N$ = 50) | 727.8 min | 8.3 min |
| Clustering into CAPs (with $K$ = 4, $P_P$ = $P_N$ = 100%) | 15.5 min | 0.1 min |
| Reconstruction | *not applicable* | 0.6 sec |
| **Total** | **755.4 min** | **21.3 min** |

Abbreviations: $K_{max}$ = maximal number of clusters to assess; $P_{CC}$ = percentage of randomly selected subsample of data; $N$ = number of folds; $K$: optimal number of clusters (CAPs); $P_P/P_N$ = fraction of positive-valued, respectively negative-valued voxels that should be kept for clustering

**Supplementary Figure 12: Co-activation pattern (CAP) maps calculated without initial PCA step based on insular seed activation using 30 healthy controls as reference population.** (**A**) Four CAPs were selected. CAPs were z-scored and only the 15% most positive and 15% most negative contributions are represented in colour (z = ± 1.04), with red representing positive contributions and blue negative contributions. Locations are displayed in Montreal Neurological Institute (MNI) standard space coordinates. (**B**) Pie charts illustrating the percentage of positive and negative contributions laying within the 17 RSN according to the convention of (Yeo et al., 2011). Seed voxels have been removed. Abbreviations: Ins = Insula, Cont = Executive control, Default = Default mode DorsAttn = Dorsal attention, Sal/VenAttn =

Salience/Ventral attention, SomMot = somatomotor, TempPar = Temporoparietal, VisCen = Central vision, VisPer = Peripheral Visual, RSN = Resting-State Network.

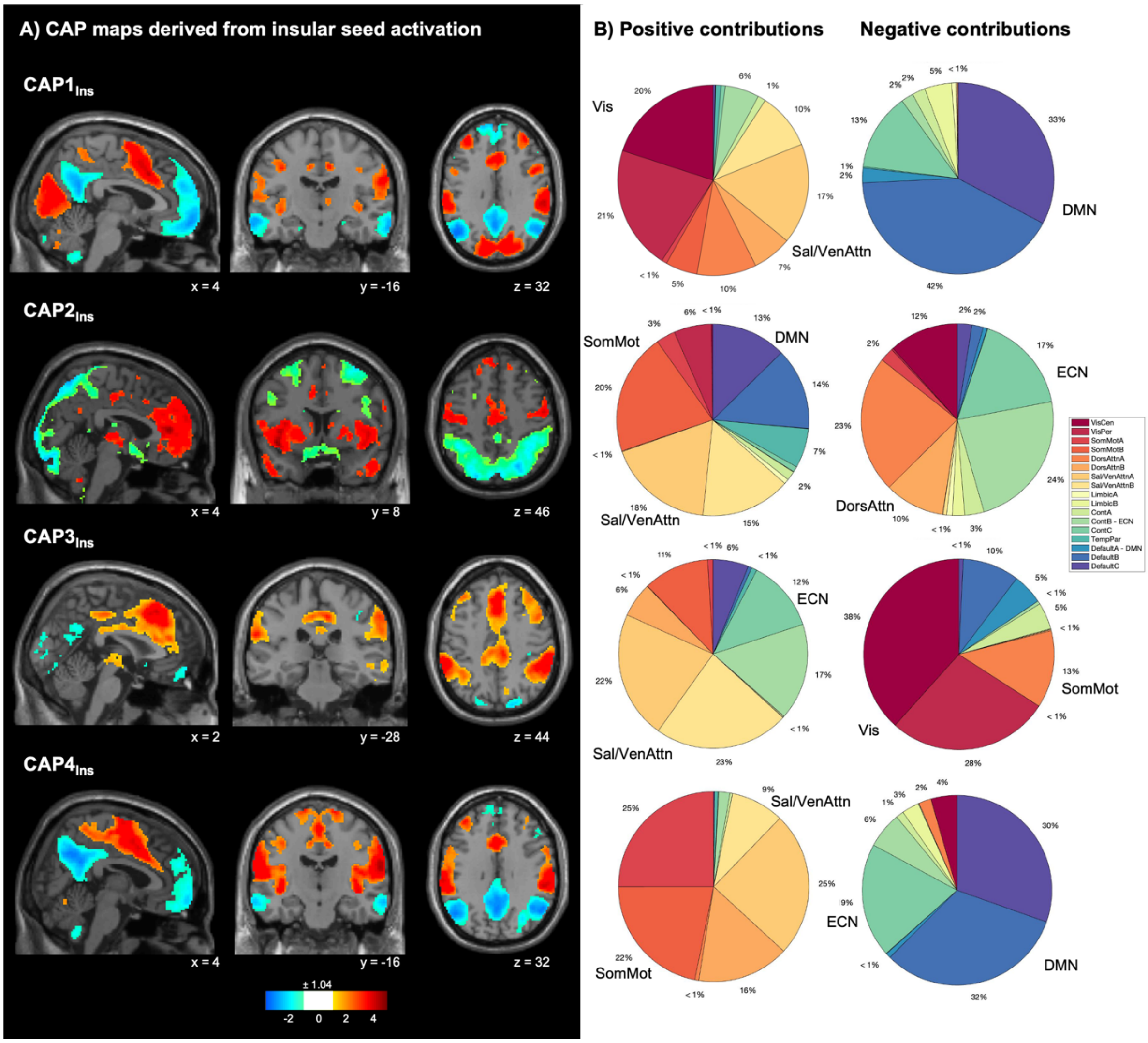

# References

Șenbabaoğlu, Y., Michailidis, G., Li, J.Z., 2014. Critical limitations of consensus clustering in class discovery. Sci Rep 4, 6207. https://doi.org/10.1038/srep06207

Yeo, Th., Krienen, F.M., Sepulcre, J., Sabuncu, M.R., Lashkari, D., Hollinshead, M., Roffman, J.L., Smoller, J.W., Zöllei, L., Polimeni, J.R., Fischl, B., Liu, H., Buckner, R.L., 2011. The organization of the human cerebral cortex estimated by intrinsic functional connectivity. J Neurophysiol 106, 1125–1165. https://doi.org/10.1152/jn.00338.2011
